# Drusen and pigment abnormality predict the development of neovascular age-related macular degeneration in Japanese patients

**DOI:** 10.1371/journal.pone.0255213

**Published:** 2021-07-27

**Authors:** Shoji Notomi, Satomi Shiose, Keijiro Ishikawa, Yosuke Fukuda, Kumiko Kano, Kenichiro Mori, Iori Wada, Yoshihiro Kaizu, Hidetaka Matsumoto, Masato Akiyama, Koh-Hei Sonoda

**Affiliations:** 1 Department of Ophthalmology, Faculty of Medical Sciences, Kyushu University, Fukuoka, Japan; 2 Department of Ophthalmology, Gunma University Graduate School of Medicine, Maebashi, Japan; 3 Department of Ocular Pathology and Imaging Science, Faculty of Medical Sciences, Kyushu University, Fukuoka, Japan; Massachusetts Eye & Ear Infirmary, Harvard Medical School, UNITED STATES

## Abstract

Drusen are known to be the important hallmark to predict the development of age-related macular degeneration (AMD). The prevalence of drusen is lower in Asians compared with Caucasians so that the role of signs constituting early AMD is not well established in Asian populations as in Western countries. In this study, we retrospectively investigated clinical characteristics and 5-year incidence of neovascular AMD (nAMD) in the fellow eye of unilateral nAMD patients. Of 296 consecutive unilateral nAMD patients who had been followed up more than 5 years, 170 typical AMD, 119 polypoidal choroidal vasculopathy, and 7 retinal angiomatous proliferation were included. To examine factors associated with nAMD occurrence in the fellow eye, drusen and pigmentary abnormality in the fellow eye were classified into 4 categories; Category 1: no or small drusen < 63 μm (37.2%), Category 2: 63–125 μm medium drusen or pigmentary abnormality (22.2%), Category 3: large drusen > 125 μm (25.0%), Category P: pachydrusen (15.5%). The mean sub-foveal choroidal thickness (SFCT) was Category 1: 276 μm, Category 2: 308 μm, Category 3: 246 μm, and Category P: 302 μm, respectively. Of note, SFCT in Category 2 and Category P was significantly larger than those of Category 3. Finally, the 5-year incidence of nAMD in the fellow eye was 32/296 (10.8%); Category 1: 0/110 (0%), Category 2: 12/66 (18.2%), Category 3: 20/74 (27.0%), and Category P: 0/46 (0%). Thus, signs of intermediate AMD (large drusen) as well as those of early AMD, especially the pigmentary abnormality, may contribute to development of bilateral nAMD in Japanese patients.

## Introduction

Age-related macular degeneration (AMD) is the leading cause of visual impairment in the elderly worldwide. Previous studies on the natural history of AMD have shown that the existence of drusen indicates a stepwise increased risk for the progression to late AMD, namely neovascular AMD (nAMD) or geographic atrophy, depending on the area, size, number, and type of drusen. Accumulations of medium drusen (63–125 μm) or the appearance of a single large druse (>125 μm) constituted the threshold to diagnose early/intermediate AMD in the Age-Related Eye Disease Study (AREDS) [1, 2]. Other studies have identified that soft drusen and/or pigmentary abnormalities is strongly associated with the development of late AMD [3–5]. Thus, not only large drusen but also medium drusen or pigmentary abnormality is involved in AMD progression.

Several clinical manifestations of nAMD seen in Asian are apparently different from Caucasians. The prevalence of nAMD is higher in Asian than those in Caucasian populations [6], while the prevalence of large soft drusen in the unaffected eye is significantly lower in Asians [7]. Recently, polypoidal choroidal vasculopathy (PCV), an AMD subtype often seen in Asian, and central serous chorioretinopathy (CSC), have been thought to share the conditions characterized by thickened choroid and dilated choroidal vessels. This has led to the idea that there might be a similar pathology, namely the “pachychoroid spectrum disease”. Such choroidal alterations are more frequently observed in Asians than Caucasians [8]. Notably, pachychoroid disease is less accompanied by typical soft drusen in the macula, however, is often associated with the so-called “pachydrusen” [9]. This newly recognized drusen often have an irregular outer contour and show a scattered wide distribution over the posterior pole, whereas typical soft drusen tend to accumulate in the central macula. Indeed, it is reported that significantly more eyes are associated with pachydrusen in Asian than in Caucasian population [10]. In contrast, reticular pseudodrusen, also known as subretinal drusenoid deposit (SDD), are less frequent in Asians.

Since the existence of nAMD may imply the risk of nAMD in the fellow eye, previous studies reported the prevalence of nAMD in the unaffected eye in Asians, accordingly [11–13]. Unlike AREDS, most population-based or hospital-based studies in Asians have defined the drusen as larger than 125 μm. Hence, the evidence related to medium drusen/pigmentary abnormality in the second eye’s AMD progression is still limited in Asians. Recently, it has been shown that pachydrusen is less associated with occurrence of nAMD compared with soft drusen in the fellow eye [14]. Thus, the role of pachychoroid and pachydrusen in the context of AMD progression could be an emerging issue. In this study, we categorized the size/area of drusen and presence/absence of pigmentary abnormality and investigated the 5-year incidence of neovascularization in the fellow eye of nAMD.

## Material and methods

### Patients

This retrospective study was conducted on patients newly diagnosed with nAMD in the Department of Ophthalmology of Kyushu University Hospital between January 2012 and June 2015. The patients’ information was obtained from April 8^th^ through April 30^th^ in 2020. A total of 831 consecutive patients were selected for medical record review containing patients’ ophthalmologic examinations and ocular history. The study was approved with a waiver of informed consent through the institutional review board at Kyushu University, Fukuoka, Japan (REB #26–131) and adhered to the tenets of the Declaration of Helsinki. All data was anonymized prior to data analysis. The only patients included in the analysis were with unilateral nAMD eye and treatment-naive fellow eye without any signs of nAMD, those who underwent more than 5-years follow-up. All patients were treated with anti-vascular endothelial growth factor (VEGF) therapy (ranibizumab, aflibercept, or bevacizumab). All patients underwent comprehensive ophthalmic examination at the initial presentation in both eyes including best-corrected visual acuity using the Landolt chart, slit-lamp biomicroscopy, intraocular pressure, color fundus photography covering the 45-degree posterior retina, fluorescein/indocyanine-green angiography (FA/ICGA), near-infrared reflectance, fundus autofluorescence (HRA-II; Heidelberg Engineering, Dossenheim, Germany), and spectral-domain OCT (SD-OCT) (Spectralis HRA + OCT) including enhanced depth imaging (EDI) OCT. At every follow-up, all examinations except angiography were performed. When the new nAMD onset in fellow eyes were suspected, FA/ICGA were performed. The subtype of nAMD, typical nAMD (tAMD), PCV, and retinal angiomatous proliferation (RAP) was comprehensively diagnosed by the findings of funduscopy, angiography, and OCT. Typical nAMD was characterized by the presence of exudative changes due to choroidal neovascularization (CNV) on FA and ICGA. The diagnosis of PCV was based on the presence of polypoidal dilatations revealed by ICGA and a sharp peak of pigment epithelium detachment (PED) on OCT images (S1 Fig). The diagnosis of RAP was based on the cystic retinal edema on OCT images, intraretinal hemorrhage, and retinal-retinal/retinal-choroidal vascular anastomoses.

### Imaging analysis

**Fig 1:**
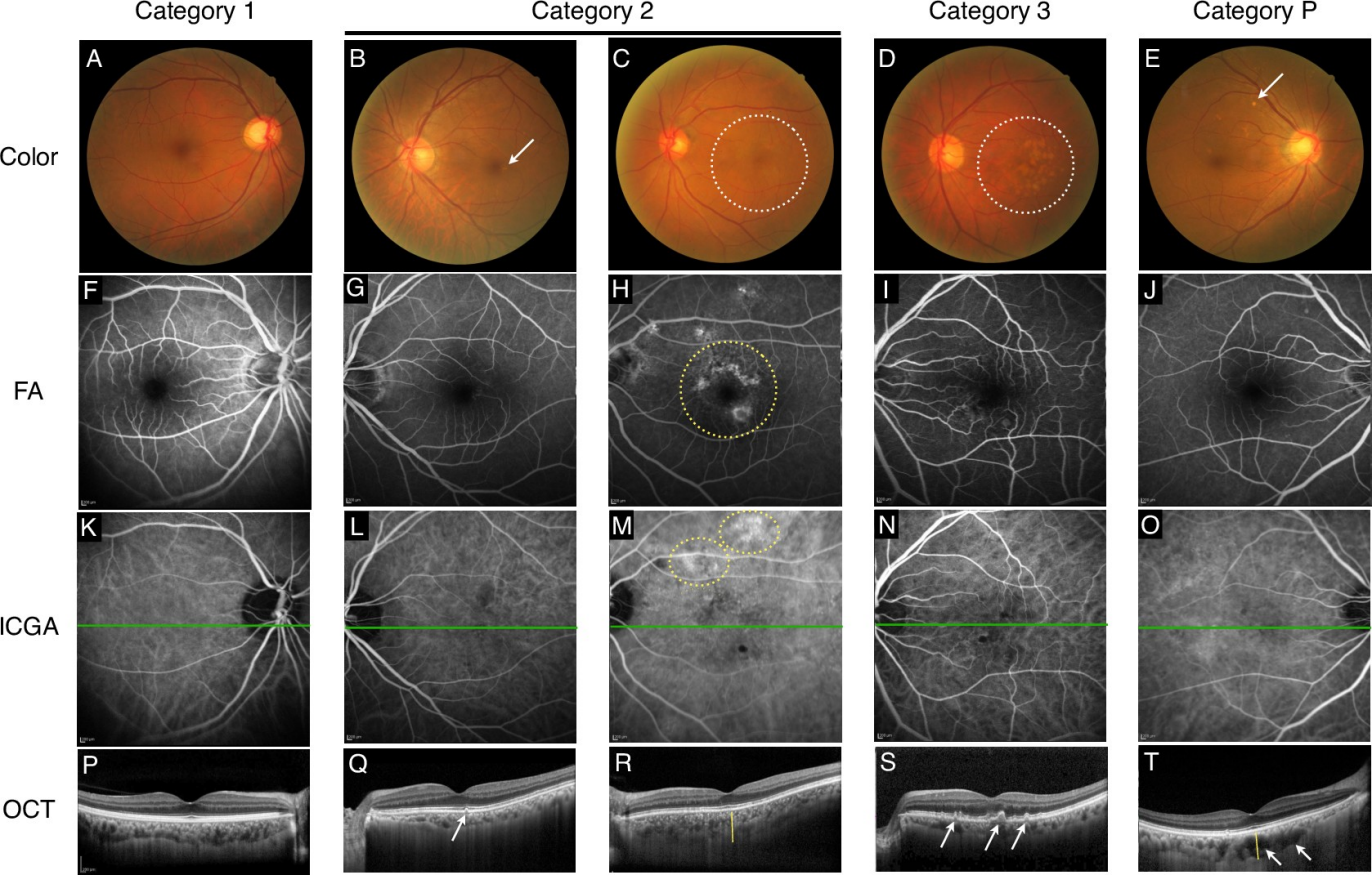
Classification of fellow eye’s conditions with multimodal imaging findings. Representative images of fundus photograph (A-E), FA (F-J), ICGA (K-O), and OCT (P-T) for each category. Representative images for Category 1 (A, F, K, and P) showed neither drusen > 63 μm nor pigmentary abnormality. Representative images for Category 2 (B, G, L, and Q) accompanied by 63–125 μm medium drusen (See an arrow in B). Note the drusen detectable by OCT as well (an arrow in Q). Representative images for Category 2 (C, H, M, and R), associated with pigmentary abnormality (See a circle in C). The RPE abnormality was revealed by the finding of window with FA (a yellow circle in H). Concurrent choroidal vascular hyperpermeability was detected by ICGA (yellow circles in M). Note that thick choroid presented (as indicated by a yellow line in R) and that sub-RPE deposits was not seen (R). Representative images for Category 3 (D, I, N, and S); soft drusen lager than 125 μm accumulating on the macula (a circle in D). OCT detected dome-shaped elevations of RPE (arrows in S). Representative images for Category P (E, J, O, and T), presenting pachydrusen as an isolated large drusen outside the macular area (an arrow in E). Note that pigmentary abnormality or macular drusen was not observed. Dilated choroidal vessels (pachyvessels; arrows in T) and thick choroid (as illustrated by a yellow line in T) was observed. Horizontal green line (K, L, M, N, and O) represents where OCT scans cross.

The conditions of fellow eyes were classified into 4 groups according to the size/location of drusen and pigmentary abnormality (Fig 1). The size/location of drusen were determined using color fundus photographs and SD-OCT. Pigmentary abnormality was defined by color fundus photography as well as FA images indicating window defect. The conditions of fellow eyes were classified into 4 categories according to the AREDS as follows. Category 1: small drusen (<63 μm). Category 2: either intermediate drusen (63–125 μm) or pigmentary abnormality. In addition, several small drusen (<63 μm) over an area of 125 μm diameter circle. Category 3: at least 1 large (>125 μm) druse or over 20 intermediate drusen. Category P: pachydrusen. Drusen was diagnosed when yellowish-white sub-RPE deposits observed in the macular area; 3-mm diameter circle centered on the fovea, which was revealed by color fundus photographs and OCT images. SDDs were diagnosed whitish deposits were observed on color fundus photographs and infrared reflectance images that corresponded to material accumulated in the subretinal space above the RPE on OCT images. Pachydrusen often had an irregular outer contour, show a scattered distribution over the posterior pole, may aggregate around the optic nerve, and occur in isolation or in groups of only a few drusen as previously described [9]. Eyes with both pachydrusen and drusen/pigmentary abnormality that correspond to Category 2 or 3 were categorized as Category 2 or 3 and excluded from Category P. The classification was confirmed by three independent graders (S. N., S. S., and K. K.) who was masked to the diagnosis of the fellow eye. Occurrence of nAMD in the fellow eye was diagnosed when there was evidence of CNV associated with serous PED, intra/sub-retinal fluid, or intra/sub-retinal hemorrhage. The SFCT was measured from the outer surface of the RPE band to the inner surface of the choroidal-scleral interface under the fovea on EDI-OCT images.

### Statical analysis

Differences among the categories and AMD subtypes were analyzed using Fisher exact test or 1-way analysis of variance (ANOVA) followed by Tukey-Kramer HSD test. Time-to-event endpoint was analyzed using the Kaplan-Meier method and log-rank test. Age and category were tested whether they were factors independently associated with nAMD occurrence using the Cox proportional hazard regression model. *P* < 0.05 was considered statistically significant.

## Results

### Clinical characteristics of the enrolled patients

A total of 831 patients who were newly diagnosed with unilateral nAMD and received anti-VEGF therapy between January 2012 and June 2015 were screened for the study.

**Fig 2:**
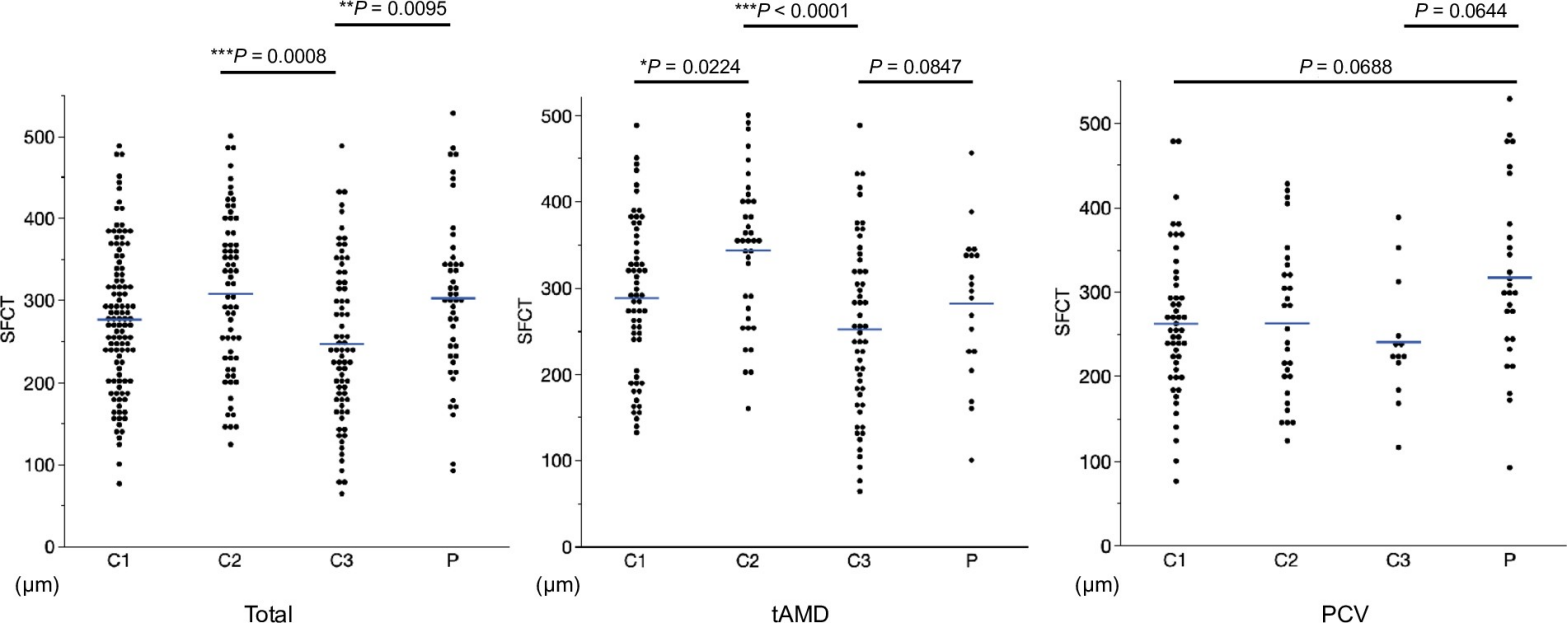
Scatter plots for the fellow eye’s SFCT in each category. Both Category 2 and Category P show significantly higher SFCT than Category 3 (*P* = 0.0008 and *P* = 0.0095, respectively). Similarly, SFCT in Category 2 was significantly thicker than Category 3 (*P* < 0.0001) in the fellow eye of tAMD. SFCT in Category P showed a trend to be higher than Category 1 or Category 3 in the fellow eye of PCV (*P* = 0.0644 and 0.0688, respectively). Tukey-Kramer HSD test. Horizontal blue bars indicate the mean SFCT in each group. *P < 0.05, **P < 0.001, ***P < 0.0001.

Among those patients, 297 patients who had been followed beyond 5 years were enrolled in the study. Clinical characteristics of enrolled unilateral AMD patients and classification of the fellow eyes are summarized (Table 1). Typical nAMD, PCV, and RAP accounted for 57.4% (n = 170), 40.2% (n = 119), and 2.4% (n = 7) of total cases, respectively. The mean (± standard deviation; SD) age of the patients at the first visit was 71.6 (± 8.4) years and there were 198 (66.9%) males and 98 (33.1%) females. The number of fellow eyes were Category 1: 110 (37.2%), Category 2: 67 (22.2%), Category 3: 74 (25.0%), and Category P: 46 (15.5%), respectively. Their mean age was significantly differed across categories (*P* = 0.0004). The proportions of categories were significantly different among the first eye’s AMD subtypes. Category 3 included less PCV and Category P involved more PCV (Table 1). The mean SFCT in each category are summarized (Table 2) and the scatter plots are illustrated (Fig 2). The SFCT was significantly differed among categories (*P* < 0.0005).

**Table 1:** Clinical characteristics of the enrolled unilateral nAMD patients and categorization in the fellow eyes.

Characteristics	Category 1	Category 2	Category 3	Category P	*p*
*n*	110	66	74	46	
Age (years), mean ± SD	69.7 ± 9.3	72.2 ± 7.7	74.6 ± 8.0	69.9 ± 6.5	0.0004
Sex (M:F), *n*	74 : 36	50 : 16	48 : 26	26 : 20	0.1932
nAMD subtype, *n*					
tAMD	60	37	54	19	0.0050
PCV	50	29	13	27	< 0.0001
RAP	0	0	7	0	NA

Age was compared with Tukey and Kramer HSD test. Sex and the ratio of the first eye’s AMD subtype (tAMD, PCV, or RAP) were analyzed by Fisher’s exact test.

**Table 2:** SFCT in each category according to the first eye’s AMD subtype.

First eye’s nAMD subtype	Category 1	Category 2	Category 3	Category P	*p*
tAMD	287 ± 89	343 ± 86	252 ± 102	281 ± 86	0.0001
PCV	262 ± 86	262 ± 90	240 ± 74	317 ± 108	0.0343
RAP	−	−	218 ± 99	−	NA
Total	276 ± 88	308 ± 96	246 ± 96	302 ± 100	0.0003

Mean ± SD of SFCT (μm) in the fellow eye were compared with 1-way ANOVA.

### Incidence of nAMD and categories in the fellow eye

**Fig 3:**
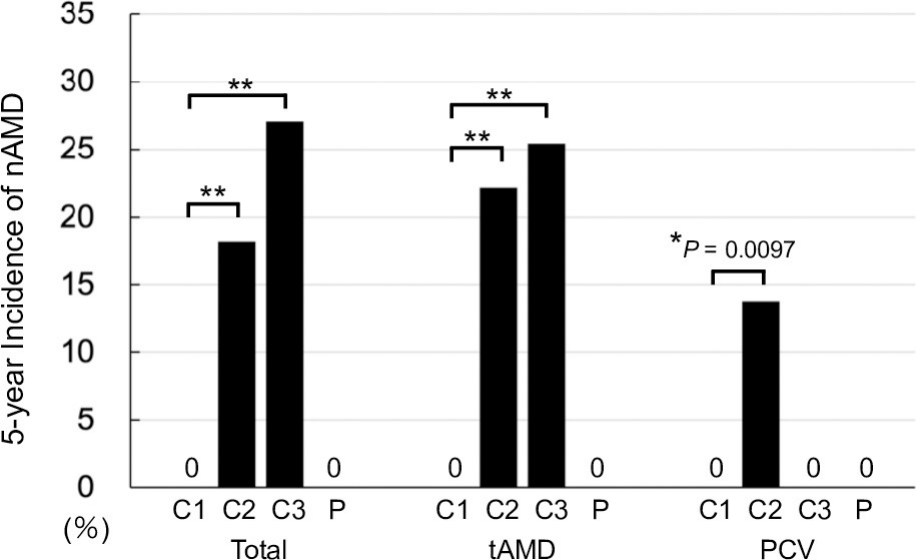
Five-year incidence of nAMD in each category according to the first eye’s AMD subtypes. **P < 0.0001. *P < 0.01. Fisher’s exact test.

**Fig 4:**
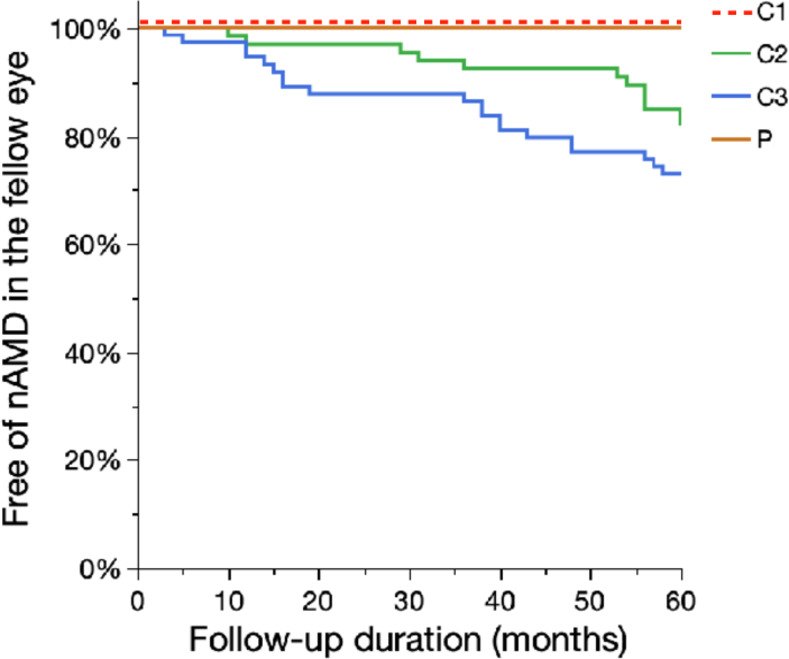
Kaplan Meier curve for nAMD-free periods in each category. The occurrence of nAMD in the fellow eye was used as an endpoint. *P* < 0.0001. Log-rank test.

Five-year cumulative incidence of nAMD in the fellow eye were 33/297 (11.1%); according to each category, Category 1: 0 of 110 (0%), Category 2: 12 of 66 (18.2%), Category 3: 20 of 74 (27.0%), and Category P: 0 of 43 (0%) (Table 3 and Fig 3). Free of nAMD period and follow-up duration are illustrated using Kaplan-Meier method (Fig 4). Cox proportional hazard model showed that category but not age was statistically significant for the occurrence of nAMD (*P* < 0.0001 and *P* = 0.4604, respectively; likelihood ratio test).

**Table 3:** Five-year incidence of nAMD in the fellow eye according to each category.

First eye’s nAMD subtype	Category 1	Category 2	Category 3	Category P	*p*
tAMD	0/60	8/37	14/54	0/19	< 0.0001
PCV	0/50	4/29	0/13	0/27	0.0091
RAP	0/0	0/0	6/7	0/0	NA
Total	0/110	12/66	20/74	0/46	< 0.0001

The occurrence of nAMD was analyzed by Fisher’s exact test.

### Incidence of nAMD in pigmentary abnormality

Since Category 2 included pigmentary abnormality and/or intermediate drusen, we clarified the proportion of intermediate drusen and pigment abnormality in Category 2. Of 66 eyes in Category 2, there were 16 eyes with intermediate drusen alone without pigmentary abnormality, in which only 1 eye developed nAMD (6.3%). Among the other 50 eyes with pigmentary abnormality, nAMD had occurred in 12 eyes (24.0%).

### Subcategories for pachydrusen

Since we classified eyes with both pachydrusen and drusen/pigment abnormality in the macular area into Category 2/3, we examined how many eyes with pachydrusen were included in Category 2/3. Sixty-one eyes with pachydrusen were categorized into Category 2/3 because of having pigmentary abnormality and/or intermediate/large drusen. Of those 60 eyes, 8 developed nAMD (Table 4). Across all the categories, 8 of 106 (7.5%) eyes with pachydrusen and 24 of 190 (12.6%) eyes without pachydrusen developed nAMD.

**Table 4:** The prevalence of pachydrusen in Category 2/3 and 5-year incidence of nAMD in the presence or absence of pachydrusen.

Pachydrusen	Category 2	Category 3	*p*			
Positive	22	38	0.0402			
Negative	44	36				
Total	66	74				
5-year incidence of nAMD						
Pachydrusen	Category 1	Category 2	Category 3	Category P	Total	*p*
Positive	0/0	2/22	6/38	0/46	8/106	0.2412
Negative	0/110	10/44	14/36	0/0	24/190	

The proportion of pachydrusen in Category 2/3 and 5-year incidence of nAMD with/without pachydrusen was compared by Fisher’s exact test.

**Fig 5:**
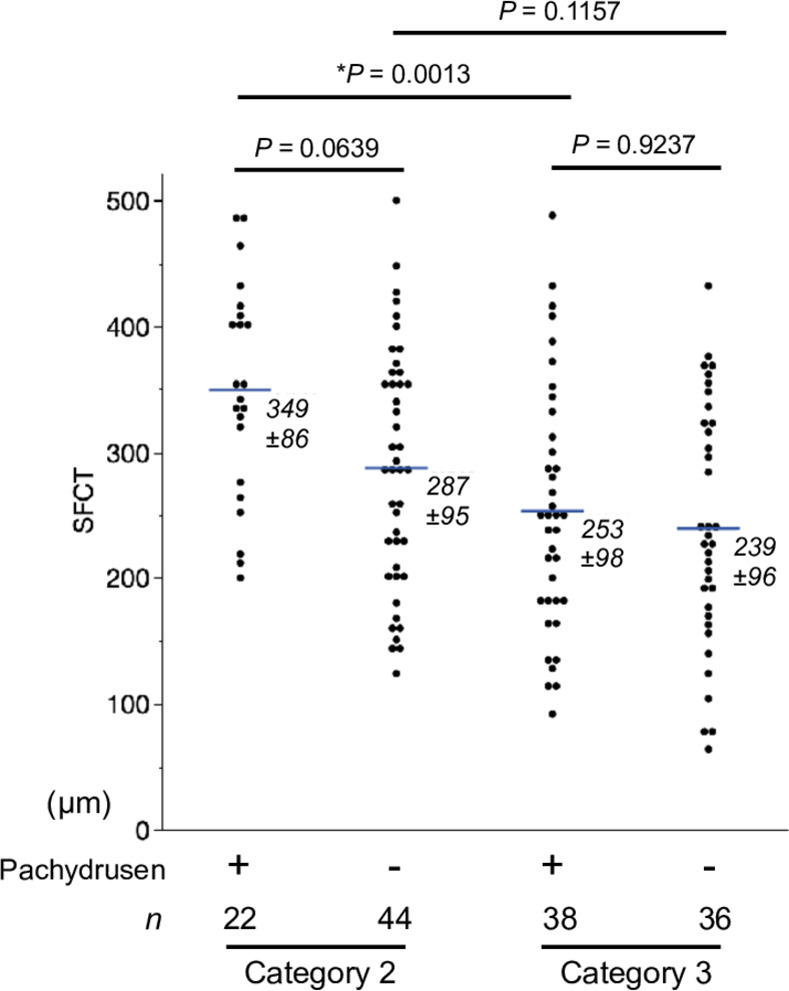
Scatter plots for SFCT of Category 2/3 in the presence or absence of pachydrusen. Horizontal blue bars indicate the mean SFCT in each group (SDs are numerically shown). *P < 0.05. Tukey-Kramer HSD test.

Additionally, we examined the association of pachydrusen in Category 2/3 and choroidal thickness. Eyes with pachydrusen in Category 2 showed a trend to have larger SFCT compared to those without pachydrusen (*P* = 0.0639), but such a trend was absent in Category 3 (Fig 5). Mean SFCT in Category 2 was significantly larger than those in Category 3 in pachydrusen-positive group (*P* = 0.0013) and showed a trend to be larger than those in Category 3 in pachydrusen-negative group (*P* = 0.1157). Although the number is limited, pachydrusen in Category 2 were accompanied by notably thick choroid (349 ± 86 μm, *n* = 22).

## Discussion

In this study, we investigated the 5-year incidence of nAMD in the fellow eye of nAMD. All the 297 patients who had unilateral nAMD with treatment-naive fellow eyes were followed up over 5 years. The ratio of AMD subtype (tAMD, PCV, or RAP) in the first eye were similar to previous reports in Asians [13–16]. The fundus conditions of fellow eyes were categorized according to the size/location of drusen and the presence/absence of pigmentary disturbance, similar to AREDS [1, 2]. Of note, the typical soft drusen larger than 125 μm as well as the presence of medium drusen/pigmentary abnormality showed higher rates of nAMD occurrence, compared to the other categories that are not accompanied by such macular lesions. Overall, our results provide compliment to previous reports showing that fellow eye’s soft drusen contribute to the development of bilateral nAMD in an Asian population. Furthermore, signs of early AMD such as medium drusen and/or pigmentary abnormality, which is equivalent to the AREDS Category 2, is the important hallmark for nAMD occurrence as well.

Several population-based or hospital-based study have revealed the prevalence of drusen/early AMD and late AMD in Asians [13–23]. Still, there are limited knowledge on the relationship between the early RPE alterations (other than typical soft drusen) and the future risk for nAMD. In this study, we classified the size of drusen and pigmentary abnormality in the fellow eye according to AREDS and found that not only Category 3 (drusen larger than 125 μm) but also Category 2 (medium drusen or pigmentary abnormality) could contribute to AMD progression. Lee *et al*. investigated the fate of drusen larger than 125μm in the fellow eye and revealed that the incidence of nAMD was higher when soft drusen combined with SDD compared with soft drusen alone [13]. The incidence of nAMD in Category 1 in this study have resulted in quite lower than that of no significant drusen in the study by Lee *et al*. The reason accounting for the difference might be due to categorization. We classified intermediate drusen (63–125 μm) and pigmentary abnormality into Category 2, whereas Lee *et al*. defined them as no significant drusen. In this study, the incidence of nAMD in Category 2 (medium drusen or pigmentary abnormality) was significantly higher than Category 1. Regarding this difference, one might suggest a possibility that the fellow eyes developing nAMD in previous studies also might have had medium drusen or pigmentary abnormality.

In this study, the fellow eyes of tAMD more frequently developed nAMD compared to those of PCV. Our results indicate that Category 2 or 3 in the fellow eye of tAMD could be the risk factor for nAMD as well. In contrast, Sasaki *et al*. reported that pigmentary abnormality in the fellow eye of PCV could be the early sign to develop nAMD [16]. There might have been difference in diagnostic criteria for PCV between the previous and the current study. Currently, most investigators base the diagnosis of PCV on ICGA findings, namely the presence of polypoidal dilatations [24]. However, the ratio of PCV in total AMD cases can differ among the literatures; a prevalence of PCV ranged 22–62% in Asians [25], indicating that diagnosis of PCV might not have been consistent among facilities. In this study, three retinal specialists confirmed the presence of typical polypoidal lesion revealed by ICGA to diagnose PCV. The other Type 1 CNV that did not show the polyp lesion by ICGA was diagnosed as tAMD (S1 Fig). Consistent with our results, Lee *et al*. reported that the 5-year incidence of nAMD in the fellow eye of PCV is 8.2%. In addition, Pang *et al*. suggested that PCV is not a distinct entity in itself but rather a manifestation of longstanding Type 1 CNV [26]. Hence, it is still unclear whether there is a threshold to distinguish PCV and the other Type 1 CNV.

Upon the classification in this study, it was shown that Category 2 in the fellow eye is significantly associated with thicker choroid than Category 3. Our results indicated that both Category 2 and 3 are the risk factor to develop nAMD in the fellow eye, whereas their choroidal thickness was significantly different. Recently, Sasaki *et al*. demonstrated that choroidal thickness was positively associated with the presence of pigmentary abnormalities in a Japanese population [27]. Our results were in line with the report by Sasaki *et al*. In other previous studies, soft drusen developing bilateral AMD were associated with thin choroid both in Asians and Caucasians [10, 23, 28]. In this study, the characteristics of choroidal thickness seem consistent with those previous reports. Moreover, it has been shown that the condition of Category 2 also confers the risk for progression of bilateral AMD in Asians. As described in the results, Category 2 that developed nAMD was mostly pigment abnormality rather than intermediate size drusen. Hence, Category 2 or pigment abnormality might be associated with pachychoroid-related pathology, which differs from drusen-driven neovascularization in AMD.

We also investigated the fate of “pachydrusen” and found that it did not result in any occurrence of nAMD unless they are accompanied by pigmentary abnormality or macular drusen. Similar to other previous reports [13, 23], pachydrusen is associated with thick choroid, but not the risk factor to develop nAMD. We defined Category P as the conditions presenting pachydrusen but not accompanied by any lesion for Category 2/3. In this study, the incidence of nAMD in Category P resulted in lower than previous studies [13, 29], possibly due to this difference. Indeed, 7.5% of the eyes accompanying both pachydrusen and pigment abnormality/macular drusen developed nAMD (Table 4). Hence, our results indicated that pachydrusen with macular drusen or pigmentary abnormality could develop nAMD, however, none of eyes with pachydrusen alone were associated with nAMD within 5 years.

There are some limitations in this study that this is a single-center retrospective study and that the enrolled patients who were referred to the hospital were Japanese only, not including other Asian patients. Choroidal thickness was assessed by manual measurement using EDI-OCT images crossing the fovea. Although with those limitations, our study has indicated that not only large soft drusen but also pigmentary abnormality may be the risk for nAMD development, which was likely associated with thick choroid.

## Supporting information

S1 Fig Representative FA/ICGA and OCT images in PCV and non-PCV occult CNV. In PCV, the polys presented as focal hyperfluorescent spots in FA/ICGA (A and B) with a sharp PED peak detected by OCT (an arrow in C). In non-PCV occult CNV, such findings for typical polyps were absent (D-F). Horizontal green line (B and E) represents where OCT scans cross.
(PDF)
