# Supplementary figures and images for: Drusen and pigment abnormality predict the development of neovascular age-related macular degeneration in Japanese patients
Source: PLoS One. 2021 Jul 27;16(7):e0255213. doi: 10.1371/journal.pone.0255213 (PMC8315508; doi:10.1371/journal.pone.0255213)

S1 Fig

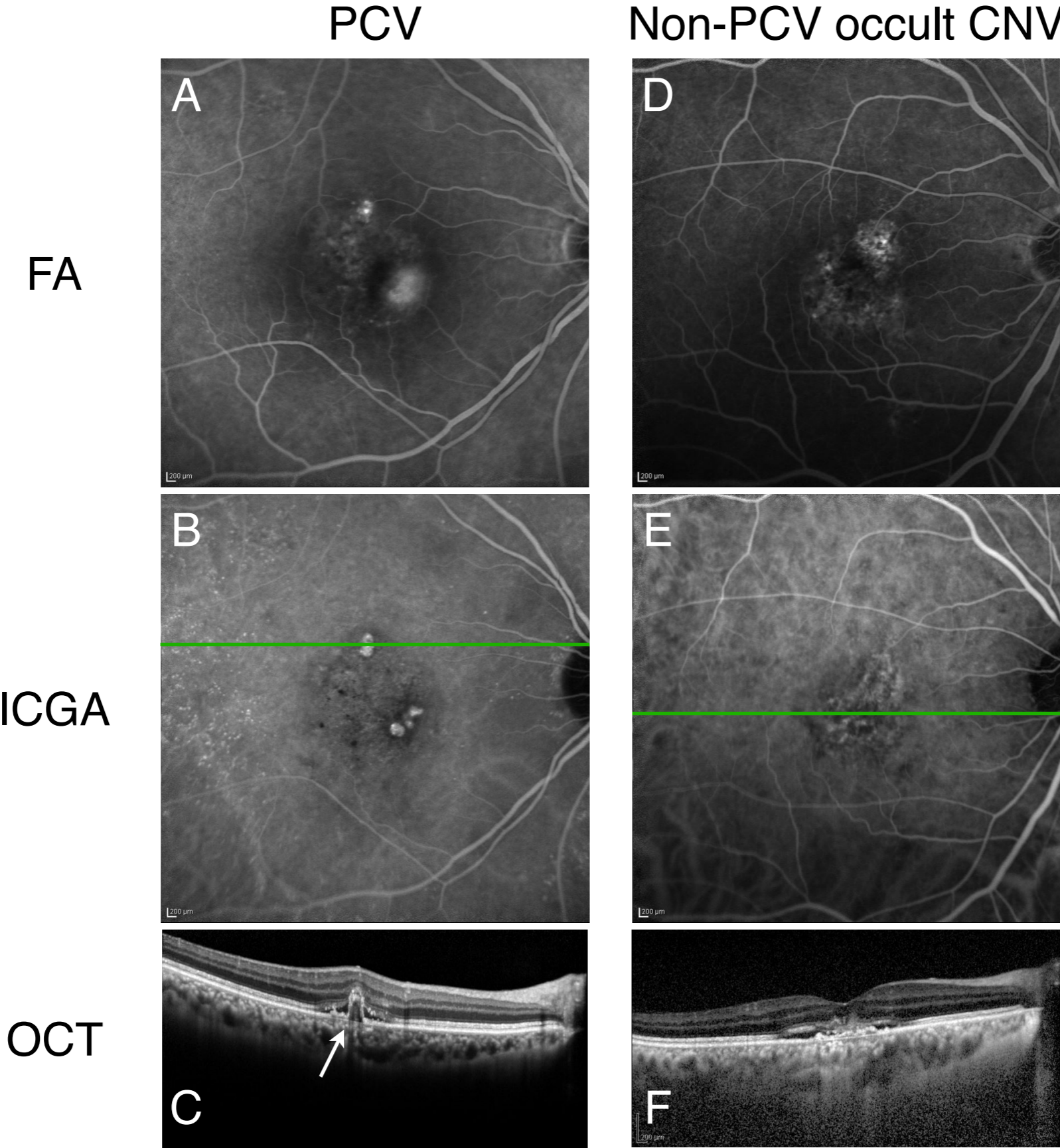

Supplement: S1 Fig — In PCV, the polys presented as focal hyperfluorescent spots in FA/ICGA (A and B) with a sharp PED peak detected by OCT (an arrow in C). In non-PCV occult CNV, such findings for typical polyps were absent (D-F). Horizontal green line (B and E) represents where OCT scans cross. (PDF) [file pone.0255213.s001.pdf]
